# Extensive Analysis of miRNA Trimming and Tailing Indicates that AGO1 Has a Complex Role in miRNA Turnover

**DOI:** 10.3390/plants10020267

**Published:** 2021-01-30

**Authors:** Axel J. Giudicatti, Ariel H. Tomassi, Pablo A. Manavella, Agustin L. Arce

**Affiliations:** Instituto de Agrobiotecnología del Litoral (CONICET-UNL), Cátedra de Biología Celular y Molecular, Facultad de Bioquímica y Ciencias Biológicas, Universidad Nacional del Litoral, 3000 Santa Fe, Argentina; axel.giudicatti@gmail.com (A.J.G.); ahtomassi@santafe-conicet.gov.ar (A.H.T.); pablomanavella@ial.santafe-conicet.gov.ar (P.A.M.)

**Keywords:** miRNA trimming, miRNA tailing, ARGONAUTE 1, miRNA turnover, *Arabidopsis thaliana*

## Abstract

MicroRNAs are small regulatory RNAs involved in several processes in plants ranging from development and stress responses to defense against pathogens. In order to accomplish their molecular functions, miRNAs are methylated and loaded into one ARGONAUTE (AGO) protein, commonly known as AGO1, to stabilize and protect the molecule and to assemble a functional RNA-induced silencing complex (RISC). A specific machinery controls miRNA turnover to ensure the silencing release of targeted-genes in given circumstances. The trimming and tailing of miRNAs are fundamental modifications related to their turnover and, hence, to their action. In order to gain a better understanding of these modifications, we analyzed *Arabidopsis thaliana* small RNA sequencing data from a diversity of mutants, related to miRNA biogenesis, action, and turnover, and from different cellular fractions and immunoprecipitations. Besides confirming the effects of known players in these pathways, we found increased trimming and tailing in miRNA biogenesis mutants. More importantly, our analysis allowed us to reveal the importance of ARGONAUTE 1 (AGO1) loading, slicing activity, and cellular localization in trimming and tailing of miRNAs.

## 1. Introduction

MicroRNAs are small 20–24 nt RNAs involved in a wide variety of processes, from development to stress responses. In plants, miRNA biogenesis begins with the transcription of a MIR gene by the RNA polymerase II. The resulting primary transcript, also called pri-miRNA, folds back into a hairpin structure, which is recognized and cleaved by Dicer-like 1 (DCL1) [1,2]. In turn, the release of the mature miRNA/miRNA* duplex may involve two or more cuts, with the processing direction dependent on the pri-miRNA secondary structure [3,4,5]. DCL1 accuracy and efficiency in releasing the precise miRNA duplex depends mainly on two core partners: HYPONASTIC LEAVES1 (HYL1) and SERRATE (SE) [2]. Within the processing complex, HYL1 activity is controlled by its phosphorylation state. C-TERMINAL DOMAIN PHOSPHATASE-LIKE 1 and 2 (CPL1 and CPL2, respectively) [6,7] and the Protein Phosphatase 4 (PP4)/Suppressor of MEK 1 (SMEK1) [8] phosphatases activate HYL1, while SnRK2 and MITOGEN ACTIVATED PROTEIN KINASE 3 (MPK3) were proposed to inactivate it [9,10]. CPL1 also dephosphorylates REGULATOR OF CBF GENE EXPRESSION 3 (RCF3), which in turn also participates in the biogenesis of miRNAs [11,12,13]. Several other proteins, such as TOUGH (TGH), DAWDLE (DDL), Negative on TATA less2 (NOT2), and the Cap-Binding complex, among others, also interact with the core DCL1/HYL1/SE complex early during miRNA biogenesis to fine-tune miRNA processing [2,14,15].

After the miRNA duplex is generated, HUA ENHANCER1 (HEN1) is recruited to the complex, it displaces SE and 2′-O-methylates the 3′ end of both miRNA strands [16]. This methylation step protects the miRNA from degradation and it is required for their accumulation; when it is lacking, miRNAs undergo trimming of the 3′ end and polyuridylation, leading to miRNA turnover [17]. Then, one of the miRNA strands, the guide strand, is loaded into an ARGONAUTE (AGO) protein, while the remaining, called passenger miRNA, is normally degraded. AGO1 is the main protein component of the RNA-induced silencing complex (RISC) acting in the miRNA pathway. It has been recently shown that AGO1 loading with a miRNA occurs in the nucleus, probably with the assistance of CONSTITUTIVE ALTERATIONS IN THE SMALL RNAS PATHWAYS 9 (CARP9), turning AGO1 into a shuttle that helps export miRNAs to the cytoplasm [18,19]. Previously, the export of miRNAs was attributed to HASTY (HST), the plant ortholog of the human Exportin-5 involved in pre-miRNA export, a function that was recently refuted, positioning HST action during early stages of the miRNA pathway [20,21].

Once in the cytoplasm, miRNA-loaded AGO1 recognizes, through sequence complementarity, target mRNAs and triggers their silencing either by slicing or translation inhibition. AGO1 subcellular localization is important not only for miRNA export from the nucleus but also for the miRNA-loaded AGO1 to exert its repressive action. AGO1 was found in microsomal fractions, which indicated that it associates with membranes [22], and it was later found that it mediates translation inhibition in the rough endoplasmic reticulum [23]. It has been also shown that AGO1 endonuclease activity is exerted in membrane-bound polysomes [24].

AGO1-bound miRNAs are also a source of miRNA turnover that involves trimming and polyuridylation of the 3’ end. HEN1 SUPRESSOR 1 (HESO1) is a terminal nucleotidyl transferase, with a preference for uridine, capable of tailing miRNAs [25,26]. While *hen1-8* mutants showed a significant increase in trimming (also referred to as truncation) and tailing, *hen1-8 heso1-1* double mutants presented much reduced tailing, supporting the role of HESO1 in polyuridylation of mainly unmethylated miRNAs. Tu et al. [27] found another nucleotidyl transferase capable of tailing miRNAs: UTP:RNA URIDYLYLTRANSFERASE 1 (URT1). Finally, miRNA degradation is performed by SMALL RNA DEGRADING NUCLEASES (SDNs), which are 3’ exoribonucleases [28]. One of the family members, SDN1, showed specificity for non-uridylated single-stranded RNA, and its activity was reduced by miRNA methylation. Although in animals there are known 5’-to-3’ exonucleases, there is no evidence of such a degradation mechanism acting on mature miRNAs in plants [16]. Interestingly both URT1 and HESO1 present activities on AGO1-bound miRNAs and they have different substrate preferences. It has also been shown that these enzymes act synergistically and are both capable of interacting with AGO1 to trigger the turnover of AGO1-loaded miRNAs [29,30]. In fact, *hen1* mutant plants also carrying the *ago1-11* weak allele presented a reduction in both tailing and trimming, which indicates that AGO1 is important for these miRNA modifications [31]. Moreover, *ago1* mutants defective in miRNA loading, such as *ago1-42* and *ago1-49* with mutations in the MID domain [32], show overaccumulation of some miRNAs [33]. However, although these processes are related to miRNA turnover, AGO1 is also considered to have a protective effect on some loaded miRNAs, since several mutants in AGO1 show a decrease in the levels of many miRNAs [16]. Altogether, these results indicate that AGO1 has a key role in modulating miRNA turnover but that it is not fully understood.

Here, we use publicly available small RNA sequencing (sRNA-Seq) data sets, including some previously generated by our group, to analyze the degree of miRNA trimming and tailing in different miRNA-related mutant plants and cellular fractions aiming to explore the complexities of miRNA turnover. Besides confirming previous results on miRNA-turnover-related genes, our results showed that miRNA biogenesis itself has a subtle but detectable impact on miRNA trimming and tailing. Several tested miRNA-biogenesis mutants displayed alterations in the miRNA trimming and tailing profiles on top of showing reduced miRNA levels. Such a detected effect is likely a consequence of the well-known DCL1 misprocessing activity in several of these mutants, a defect that led to the accumulation of inactive miRNAs that are prone to degradation. In addition, we found that miRNAs loaded on an AGO1 mutant protein retained in the nucleus are protected from miRNA trimming and tailing. Conversely, our data showed that alterations in cytoplasmic AGO1 stability, loading, slicing, or compartmentalization activity have a deep impact in miRNA turnover. Having a better understanding of how miRNA turnover proceeds is key to getting a more complete picture of miRNA regulation and action and how each step in the miRNA biogenesis can affect the fate of the mature miRNA duplex.

## 2. Materials and Methods

### 2.1. Read Processing and Filtering

Reads from selected samples (Supplementary Table S1 and Supplementary Table S2) were downloaded from public repositories in sra format and converted to fastq format using fastq_dump (SRA-toolkit, https://trace.ncbi.nlm.nih.gov/Traces/sra/sra.cgi?view=software) Adapters at the 3′ end of reads were trimmed using cutadapt [34], and only trimmed reads were preserved. The quality before and after trimming was evaluated with FastQC [35]. The results were summarized with MultiQC for a simpler inspection [36].

### 2.2. Trimming and Tailing Analysis

Trimmed reads were mapped, using Bowtie [37], to an *Arabidopsis thaliana* genome (Athaliana_167_TAIR9 from Phytozome V10.3, https://phytozome.jgi.doe.gov) in which miRNA sequences were masked. Masking was performed using the mature guide and star miRNA coordinates (not the whole primary MIR transcript) from the Araport11 gff annotation file (with “miRNA” as feature in the Araport11_GFF3_genes_transposons.201606.gff file). Reads not mapping to this masked genome were potentially unaltered, trimmed, or tailed miRNAs and were thus further analyzed. As a consequence of this strategy, for some miRNAs with perfect sequences matching elsewhere in the genome there was a loss of reads and we decided to exclude them from the analysis (Supplementary Table S3). This included 16 guide miRNAs, few with described functions, such as miR171a-3p and miR400, and 32 miRNA*.

The resulting fastq file with non-mapping reads was processed with a simple bash scripts (zcat reads.fastq.gz|awk ‘NR%4==2′|sort|uniq-c) to reduce reads with the same sequence to counts per sequence. These count tables (Supplementary Table S4) were then processed in R [38] to evaluate trimming, tailing (any nucleotide not matching the miRNA) and combinations of these processes up to the seventh position for different miRNAs, guide and star, obtained from miRBase [39]. MiRNAs were collapsed when members of the same family had identical mature sequences. Supplementary Table S5 lists which miRNAs were considered guide miRNAs. Trimming and tailing indexes were then calculated for each miRNA on each replicate by counting the number of trimmed or tailed molecules, irrespective of the degree of trimming or tailing, and by dividing this count over the total read count for the considered miRNA (Supplementary Table S6). Index differences were then obtained by subtracting from the mean index, the average of all replicates. for a given line, genotype, fraction, etc; the mean index of the reference line (Supplementary Table S7).

### 2.3. Statistical Analysis and Graphs

The statistical significance of trimming or tailing in a line with respect to the reference line was determined by first obtaining the mean of trimming or tailing indexes between replicates for both lines and then by performing a paired t-test, pairing mean indexes for each miRNA. Then, *p*-values for all tests, divided by modification, were corrected with the false discovery rate (FDR) method across all tests, irrespective of the study grouping used in the manuscript (Supplementary Table S8). All statistical analysis and graphs were obtained in R [38]. For this, the following packages were used: ggplot2 [40], the tidyverse suit of packages [41], and gplots [42].

R scripts used to compute trimming and tailing indexes, IDs, and statistical tests are available in https://github.com/manavellalab/miRNA_trimming_tailing. 

## 3. Results and Discussion

### 3.1. Systematic Genome Wide Analysis of miRNA Trimming and Tailing in miRNA-Deficient Mutants

**Figure 1:**
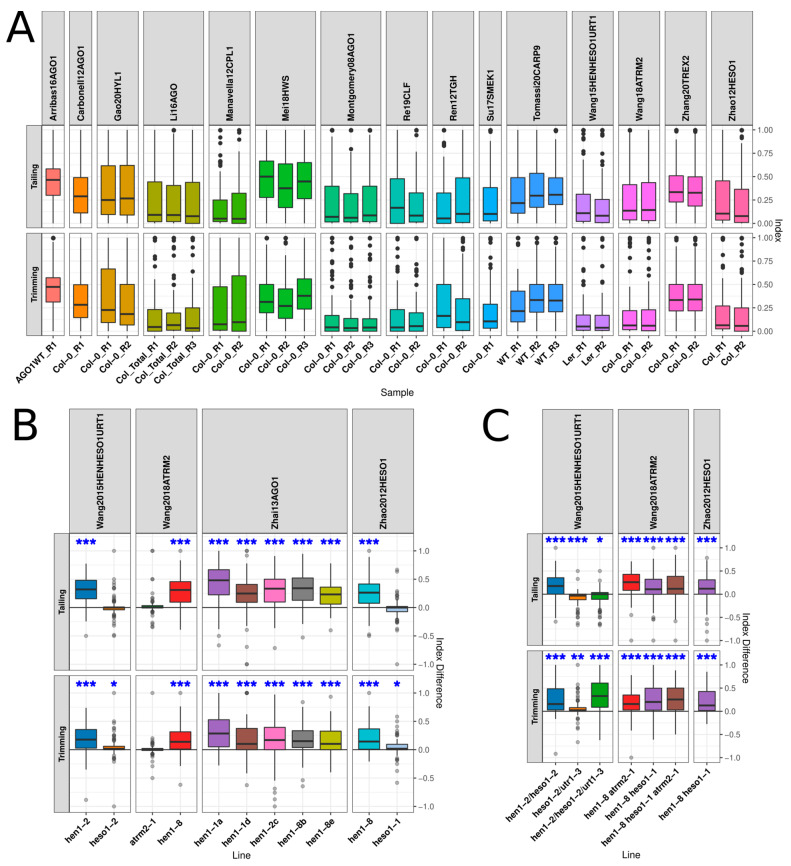
Trimming and tailing index differences enabled compensating for interstudy variability and proved capable of detecting known effects of mutating genes involved in miRNA turnover: (**A**) distribution of trimming and tailing indexes for guide miRNAs in wild type (WT) plants from the different studies analyzed, (**B**) distribution of trimming and tailing index differences (IDs) of guide miRNAs in single mutants of genes known to be involved in miRNA turnover, and (**C**) distribution of IDs of guide miRNAs in doble mutant plants of genes involved in miRNA turnover. Asterisks over the boxplots indicate the results of paired t-tests between the mean index of the line and that of the reference line, paired by miRNA; * *p*-value below 0.05, ** *p*-value below 0.01, and *** *p*-value below 0.001. Tests were grouped by the modification evaluated, trimming or tailing, and *p*-values were FDR-corrected across all studies analyzed in this work.

In order to explore the processes of miRNA trimming and tailing systematically, we analyzed small RNA sequencing studies performed using samples extracted from plants with mutations in genes encoding several key factors in the miRNA pathway (Supplementary Table S1 and Supplementary Table S2). The data were processed with a pipeline adapted from the method described by Zhao et al. [26] (see the Materials and Methods section), generating read counts associated with miRNAs with different degrees of trimming, tailing, and combinations of both. Then, for each miRNA in each sample, we computed a trimming and a tailing index, which is the proportion of trimmed or tailed reads over the total read count for the miRNA (each read counting as one, without taking into account the number of bases trimmed or added). Interestingly, when we calculated the trimming/tailing indexes among wild-type samples used as controls in the different studies, we found that the distribution of values was rather variable among studies but similar between replicates (Figure 1A). This observation probably reflects the differences in the sampled material, tissue, and/or developmental stage used in each study (Supplementary Table S1 and Supplementary Table S2). It could also be introduced by sample manipulation and library preparation. In any case, this result indicates that the experimental design when studying miRNA turnover needs to minimize all sources of variability among samples to reduce such a dispersion. Because of this issue, although statistical analyses were carried out on paired mean indexes, we generated and plotted the mean index difference (ID) as a measure unit. To compute the ID, we obtained the mean of the indexes for each miRNA in all replicates of a line and then subtracted from this value the mean index of the corresponding miRNA in the control line. In fact, the difference between paired values, the indexes, is part of the paired *t*-test that we used to evaluate significance and therefore an appropriate measure. The ID is also advantageous as it has a simple interpretation: values close to zero indicate little or no change in the modification, trimming or tailing; positive values show increases in the modification with respect to the reference line, and the opposite is the case for negative values. By doing this, distribution plots (boxplots) are easily interpreted without the need to compare distributions between a line and a reference line, which are additionally variable between studies, as shown in Figure 1A. Also, the pairing of indexes for each miRNA cannot be captured by independently plotting mean indexes for all miRNAs in each line.

As a proof of concept of our computational setting, we first analyzed mutant samples already described to display a clear effect over miRNA trimming and tailing. As expected, the distribution of the IDs confirmed the previously known effects regarding trimming and/or tailing in plants harboring mutant alleles of *HEN1*, *HESO1*, and *URT1* (Figure 1B,C). A statistically significant increase in trimming and tailing was observed in the different HEN1 single mutants, *hen1-1*, *hen1-2*, and *hen1-8*, as expected by the loss of 2’-O-methylation, which protects miRNAs, but not in single *atrm2-1* mutants (Figure 1B). Single *HESO1* mutant plants showed a slight increase in trimming and no differences in tailing (Figure 1B). The increment in miRNA tailing observed in the *hen1* mutants is partially decreased in double *hen1*, *heso1* mutants and abolished in *hen1/heso1/urt1* mutants, which is expected since these two genes are responsible for miRNA tailing. In these triple mutants, tailing is even significantly reduced with respect to wild type (WT) plants (Figure 1C). Mutations in *atrm2* did not appear to modify the trimming/tailing index of *hen1* mutants (Figure 1C). No URT1 single mutant plants were analyzed, but *urt1/heso1* double mutants had a significant increase in trimming and a decrease in tailing in the WT and in the *hen1-2* background. However, in the *hen1* background, the increase in trimming was greatly amplified (Figure 1C).

### 3.2. Mutation of Genes Encoding Proteins Acting on Early miRNA Biogenesis Stages Leads to Altered miRNA Trimming or Talling

**Figure 2:**
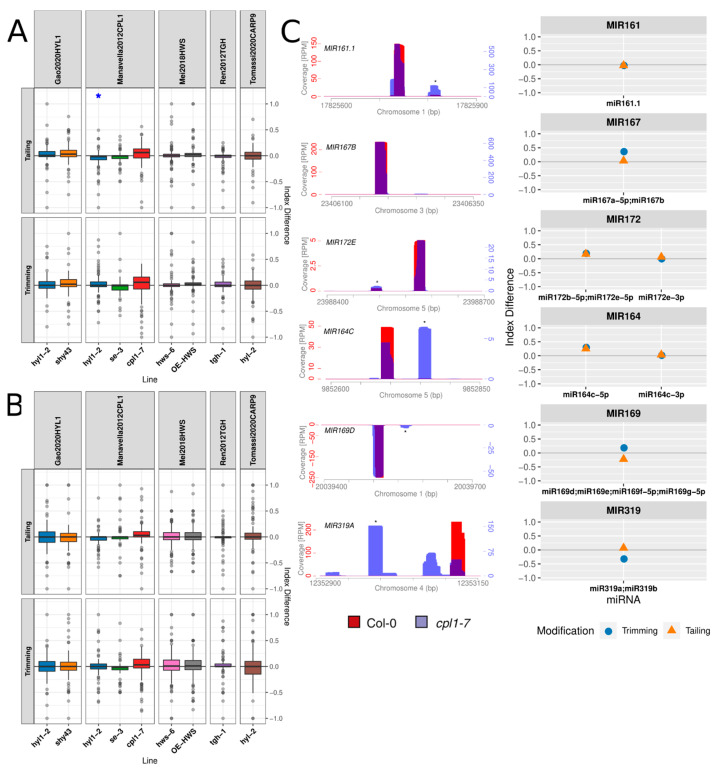
Trimming and tailing index differences of miRNAs in mutants of genes related to miRNA biogenesis: (**A**) distribution of IDs of guide miRNAs and (**B**) distribution of IDs of miRNAs*, where asterisks over the boxplots indicate the results of paired t-test between the mean index of the line and that of the reference line, paired by miRNA, and * *p*-value below 0.05. Tests were grouped by the modification evaluated, trimming or tailing, and *p*-values were FDR-corrected across all studies analyzed in this work. (**C**) Normalized coverage of small RNA reads at different MIR loci: the Y-axes are labeled on the first plot. Col-0 and *cpl1-7* coverage colors are partially transparent and their overlap is purple. MiRNA* is indicated by an asterisk. On the right of each coverage plot, the corresponding ID values for the miRNA (and the miRNA* if annotated) are presented.

The mutation of genes encoding proteins acting in the miRNA processing normally produce a reduction in the miRNA population, alteration in the miRNA/miRNA* sorting, and errors during DCL1-mediated miRNA duplex release. miRNA misprocessed species are commonly degraded as they fail to be active. To test whether mutations in miRNA biogenesis factors triggering misprocessing could also affect the fate of miRNA inducing their trimming/tailing, we repeated our analysis using small RNA sequencing data obtained from *hyl1*, *cpl1*, *se*, and *tgh* mutants. Interestingly ID calculation showed a subtle but significant difference between these mutants and control plants regarding trimming or tailing (Figure 2A). Similarly, mutations of SUPPRESSOR OF HYL1 (SHY43), also known as SOP1, a cofactor of the nucleoplasmic exosome controlling pri-miRNA stability [43], affected miRNA tailing and trimming (Figure 2A). Plants overexpressing the *HAWAIIAN SKIRT* (*HWS*) gene but not the mutants in this gene also showed altered IDs (Figure 2A). This result is in agreement with HWS function controlling miRNA abundance by likely inducing the degradation of a still unidentified miRNA-related factor [44], potentially functioning as a clearance mechanism for RISC, as was proposed for complexes with the miRNA and the target mimics [45]. When we explored the trimming/tailing indexes of the miRNA* strands in the same mutant plants, we observed normal ID values, probably as these passenger species are relatively low in abundance even when the miRNA/miRNA* strand sorting is altered (Figure 2B).

In addition to a reduction in the miRNA abundance and changes in strand sorting, it is common to observe aberrant miRNA molecules of incorrect sizes produced in some of these mutants, probably as a consequence of the DCL1 imperfect processing. To test whether these imperfect miRNA species represent cases of enhanced miRNA trimming or processing mistakes, we compared the small RNA profile and trimming/tailing index in individual miRNA loci in *cpl1* mutants, which was reported to present miRNAs of smaller sizes [6] and control plants (Figure 2C). Our analysis showed milder but detectable increment in trimming of some of the detected small species, suggesting that the aberrant molecules can be the consequence of combined misprocessing of the pri-miRNAs and trimming of the resulting molecules (Figure 2C).

### 3.3. AGO1 Loading and Slicing Activity Showed an Important Role in miRNA Turnover

**Figure 3:**
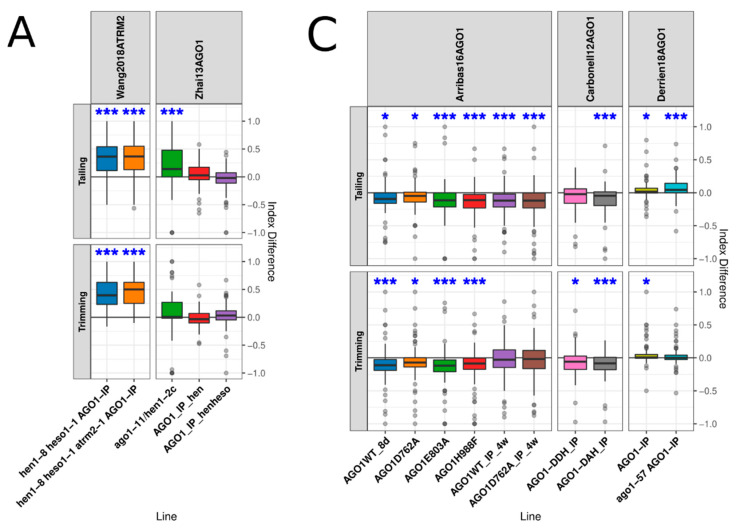

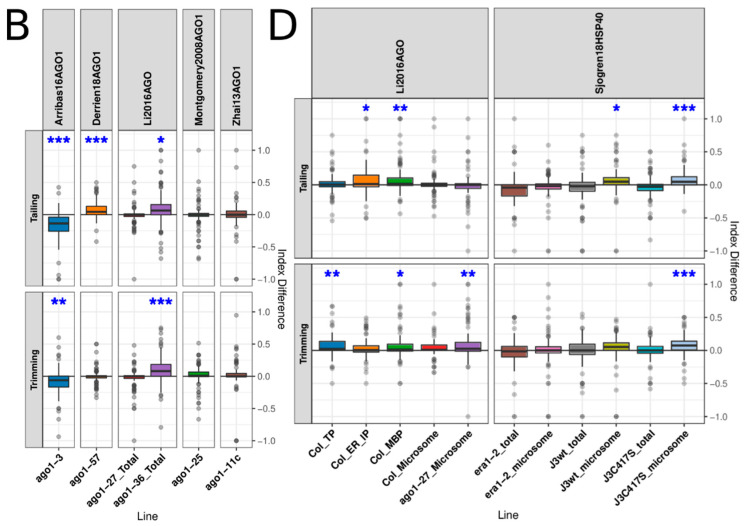
The analyses of AGO1 mutant plants, immunoprecipitations, and cellular fractions showed the relevance of this protein and its location for miRNA turnover: (**A**) distribution of guide miRNA IDs in AGO1 immunoprecipitation in mutant backgrounds of genes related to miRNA turnover, where the IDs of IPs from Zhai et al. [31] were contrasted against the sequencing of input material in the corresponding mutant background; (**B**) distribution of guide miRNA IDs in different AGO1 mutants, where the reference line for *ago1-3* was the same mutant complemented with FLAG-AGO1; (**C**) distribution of guide miRNA IDs in AGO1 IPs of WT and slicer-defective proteins, where in the AGO1 study performed by Arribas et al. [49], 8d indicates 8 days after germination, 4w indicates 4 weeks after germination, TP indicates total polysomes, and MBP indicates Microsome-Bound Polysomes and where the reference line and the rest of the samples were taken from 12 days-after-germination plants; and (**D**) distribution of guide miRNA IDs in different subcellular fractions and mutants, where in the Sjogren et al.’s [51] study, the ID was calculated with respect to different reference lines: total miRNAs were contrasted with the WT total fraction, while microsomal fractions were compared to the WT microsomal fraction. Asterisks over the boxplots indicate the results of paired t-tests between the mean index of the line and that of the reference line, paired by miRNA; * *p*-value below 0.05, ** *p*-value below 0.01, and *** *p*-value below 0.001. The tests were grouped by the modification evaluated, trimming or tailing, and *p*-values were FDR-corrected across all studies analyzed in this work.

MiRNA turnover was proposed to occur mainly when these small RNAs are bound to AGO1. As expected from a previous report, when we calculated the ID of AGO1-bound miRNAs, obtained from the sequencing of small RNAs immunoprecipitated together with AGO1 in *hen1*, *hen1/heso1*, and *hen1/heso1/atrm12* [46], we observed an increment in trimmed and tailed species, a result compatible with HESO1/UTR1 activity over AGO1-bound miRNAs (Figure 3A). However, this increment appeared to be variable since, in the results we obtained from [31], sRNA-Seq for AGO1 immunoprecipitations (IPs) in the *hen1* and *hen1/heso1* backgrounds, trimming was not statistically altered while tailing was mildly reduced in the *hen1/heso1* background (Figure 3A). This variability is not explained by the sample source as they are similar: the inflorescences of 6-week-old plants in [46] and the flowers of 5-week-old plants in [31] (Supplementary Table S1). Aiming to further explore the process of AGO1-bound miRNA trimming and tailing, we analyzed the IDs in different AGO1 mutant alleles. Three of them contained mutations in the PIWI domain of AGO1, which are not expected to affect miRNA loading [32]. The *ago1-11* mutant, which is in the Ler accession background, allowed Zhai et al. [31] to show that AGO1 slicer activity was not necessary for miRNA modifications, although it partially suppressed *hen1-2* mutants by reducing trimming and tailing in many miRNAs. The *ago1-25* mutant was shown to still associate and stabilize miR173 despite the reduction of this miRNA in the mutant [47]. The *ago1-27* allele was shown to have no important effect on global miRNA levels, to be able to load miRNAs, and to even retain some slicer activity [24], Interestingly, these three mutants displayed normal global trimming/tailing indexes (Figure 3B). As reported by the authors [31], an overall slight reduction in trimming and tailing was observed in *ago1-11/hen1* double mutants (Figure 3A) with respect to *hen1* mutants from the same study and compared to the same reference line (Figure 1B). The observations on this three AGO1 mutant alleles are compatible with the proposed model of turnover of AGO1-bound miRNAs as these mutants probably retain AGO1 loading capacity. Similar to the mutations in the PIWI domain, a point mutation within the DUF1785 domain present in the *ago1-57* allele, which renders the protein resistant to degradation by the viral P0 F-box protein [48], did not display altered trimming IDs (Figure 3B). Interestingly, it did present a significant effect on tailing. Conversely, *ago1-36*, which is a strong allele presenting a T-DNA insertion within the PAZ domain [24,32], showed both altered trimming and tailing indices (Figure 3B). The increased miRNA ID in the strong *ago1-36* allele but not in the other mutants may indicate that unloaded miRNAs are prone to spontaneous tailing and trimming even in the presence of HEN1. In contrast, the strong *ago1-3* null allele showed a significant reduction in the trimming and tailing ID when compared to the *ago1-3* mutant line complemented with an AGO1-FLAG protein used as reference line [49]. The study of miRNA trimming and tailing in AGO1 mutants with impaired loading is in order to further explore this phenomenon. Unfortunately, there are few *ago1* alleles containing mutations within the MID domain that could be predicted to affect loading [32] and no small RNA sequencing is available for them.

To further analyze the effect of miRNA loading in AGO1 in the process of tailing and trimming, we analyzed the IDs of miRNA bound to catalytically active and inactive AGO1. Carbonell et al. [50] created two tagged versions of AGO1: AGO1-DDH, the WT protein, and AGO1-DAH, a mutant protein lacking slicer activity. Then, they used them to transform *ago1* mutant plants, co-immunoprecipitate associated small RNA and sequence them. They found similar miRNA enrichment and miRNA-to-miRNA* ratios in both lines, which indicated that compromising slicer activity did not affect loading or strand selection. Surprisingly, our results showed that plants expressing the slicer-defective AGO1-DAH mutant have a marked decrease in trimming and tailing (Figure 3C). WT AGO1-DDH also showed an effect on trimming, but no effect was observed in tailing. Confirming the observation on AGO1-DAH mutants, D762A, E803A, D848A, and H988F slicer-defective AGO1 mutants [49] also showed a reduction in the trimming and tailing IDs (Figure 3C). Taken together, these results suggest that the binding of a miRNA to AGO1 is likely to protect it from spontaneous trimming and tailing but that these processes are induced upon AGO1 slicing. Supporting this scenario, when compared to the total fraction, miRNA IPs from the endoplasmic reticulum, the cytoplasmic location of AGO1-mRNA targeting, miRNAs isolated from total and membrane-bound polysomes, and the microsomal fraction showed mild but significant increases in trimming (Figure 3D) [24]. However, no significant difference in tailing was observed in this fractions. Despite *ago1-27* total small RNAs showing no difference in tailing or trimming (Figure 3B), the microsomal fraction of these mutant plants exhibited a mild but significant increase in trimming with respect to the WT total miRNAs (Figure 3D). This evidence suggests that miRNA-target slicing or at least recognition of a target-mRNA by the RISC is probably triggering miRNA turnover by trimming/tailing, perhaps as a mechanism to release and recycle AGO1 after target silencing. This is also in agreement with our previous results indicating that a prolonged AGO1–target interaction with a non-cleavable mRNA would favor AGO1 and miRNA degradation [33], which could in turn inhibit or hinder further processing of the loaded miRNA. The AGO 1 membrane association is, at least partially, dependent on farnesylation of the Heat Shock Protein 40 chaperones J3 and J2, two AGO1 interactors [51]. AGO1 levels in microsomes is reduced not only in *j3/j2* mutants but also in mutants of the farnesyl transferase ERA1. Our analysis of sRNA-Seq reads from Sjogren et al. [51] showed a mild reduction in trimming and tailing of the *era1-2* mutant in total miRNAs; no change in J3wt, the WT J3 expressed in the *j3/j2* background; and a subtle but significant reduction of tailing in lines expressing the farnesylation-deficient point mutant J3^C417S^ in the same background, which presents slightly lower levels of membrane-bound AGO1, implying that farnesylation may have a predominant role over miRNA turnover (Figure 3D). Then, we analyzed mutant microsomal fractions using the WT microsomal fraction as a reference. The *Era1-2* fraction presented decreased tailing and no change in trimming; the J3wt and J3^C417S^ fractions had a mild increase in both modifications. It should be noted, however, that the authors found both J3 proteins to be overexpressed, which in J3^C417S^ resulted in the strong induction of HSP70 and HSP90, proteins involved in RISC loading [51]. Taken together, these data indicate that the farnesylation of J3 plays a role in miRNA turnover through its effect on AGO1 membrane association.

### 3.4. AGO1 Homeostasis Had Little Effect on miRNA Turnover

**Figure 4:**
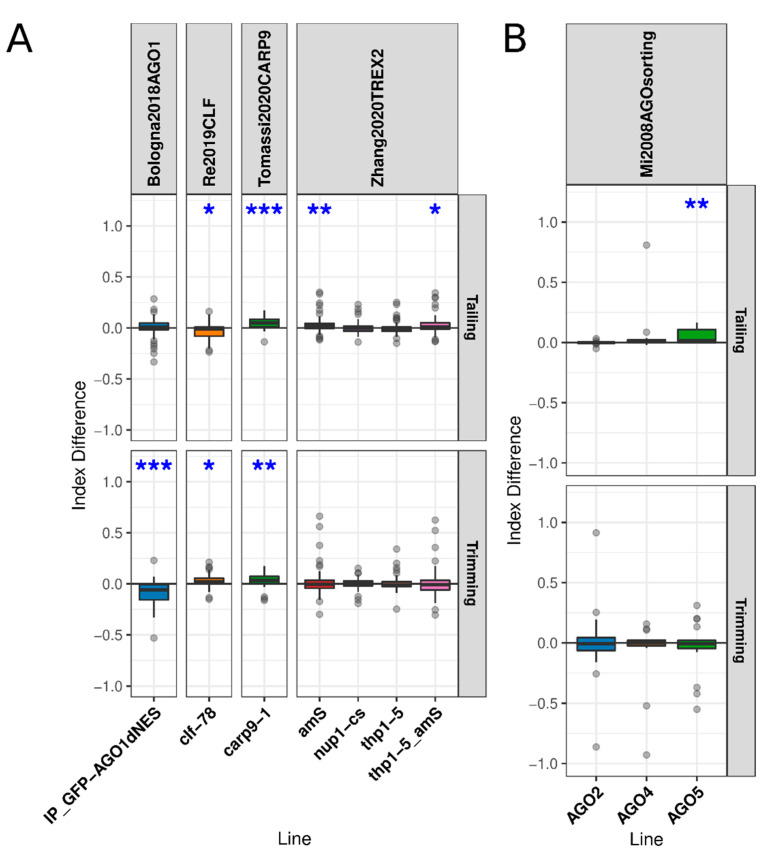
AGO1 homeostasis and the contrast with different AGO proteins: (**A**) distribution of guide miRNA IDs in mutants related to genes related to AGO1 nuclear homeostasis, loading, and miRNA export and (**B**) distribution of guide miRNA IDs in IPs of different AGO proteins using AGO1 IP as a reference, where the asterisks over the boxplots indicate the results of paired t-tests between the mean index of the line and that of the reference line, paired by miRNA. * *p*-value below 0.05, ** *p*-value below 0.01, and *** *p*-value below 0.001. The tests were grouped by the modification evaluated, trimming or tailing, and *p*-values were FDR-corrected across all studies analyzed in this work.

In recent years, novel functions were attributed to AGO1 in the nucleus of the cell. These include from DNA damage repair and transcriptional regulation to its own turnover and even miRNA export to the cytoplasm [19,33,52,53]. To explore whether these functions may affect miRNA turnover, we first evaluated the ID of miRNAs bound to a green fluorescent protein (GFP)-tagged version of AGO1 with a mutated nuclear export signal (AGO1-dNES) that forces AGO1 to be retained in the nucleus [19], using a WT-flagged AGO1 IP as reference. Interestingly, while we did not find any alteration in the tailing index, we observed a significant decrease in miRNA trimming in this fraction of the miRNAs (Figure 4A). This profile, which is the opposite to the one observed in the microsomal fraction of *ago1-27* or in *ago1-36*, suggests that nuclear AGO1-bound miRNAs are protected from degradation, a scenario compatible with the proposed spontaneous degradation of unbound miRNA and the slicing/target-dependent degradation of bound miRNAs. 

Mutations in *THP1*, core components of the TREX-2 complex, and *NUP1,* a nucleoporin protein, which were recently proposed to participate in the nuclear export of miRNAs [54], showed no changes in trimming or tailing (Figure 4A). Mild effects were only observed for tailing IDs in reporter line pSUC2: amiR-SUL (amS) and in *thp1-5*/amS double mutant (Figure 4A), which might be due to the developmental effects of amS lines. In contrast, mutant plants in the *CARP9* gene, which encodes an intrinsically disordered protein that aids AGO1 miRNA loading in the nucleus (Figure 4A) [18], presented enhanced trimming and tailing. Similarly, *CLF* mutant plants that were recently shown to affect AGO1 nuclear function and homeostasis also showed a slight increase in trimming but presented a decrease in tailing IDs (Figure 4A). Taken together, our results suggest that nuclear retention of AGO1-loaded miRNAs protects miRNA from degradation.

We also compared trimming and tailing of AGO2-, AGO4-, and AGO5-bound small RNAs [55]. AGO2- and AGO4-associated small RNAs displayed a normal trimming and tailing profile (Figure 4B). However, when we analyzed AGO5-bound miRNAs, we detected a significant increment in the tailing of associated miRNAs (Figure 4B).

## 4. Conclusions

MiRNA turnover in plants involves the processes of tailing and trimming. The enzymes directly responsible for these modifications have been described, but less is known about how these modifications occur and how they are regulated. Our systematic analysis of small RNA sequencing data confirmed the known role of these enzymes in the turnover pathway and provided evidence that many genes involved in the first steps of miRNA biogenesis affect trimming or tailing. This approach also allowed us to get a deeper picture of the role of AGO1, the main effector protein for miRNA-mediated silencing, and where and when these modifications could take place. A lack of AGO1 observed in the strong null allele *ago1-36* increases trimming and tailing of unloaded miRNAs, while loading into AGO1, observed in weak alleles, would have a protective effect. Supporting this observation, deficient nuclear miRNA loading leads to an increase in trimming and tailing. Furthermore, IPs of slicing-defective AGO1 proteins showed a reduction in trimming and tailing, indicating that target mRNA slicing, or at least target recognition, could be the event triggering these modifications. As a result, the miRNA is degraded and the RISC is free to load a new miRNA. Supporting this recycling mechanism, our previous results indicated that a prolonged AGO1 interaction with a non-cleavable mRNA would favor AGO1 and miRNA degradation [33]. Furthermore, AGO1 IP showed a mild but significant increase in trimming and tailing, and the IPs from the endoplasmic reticulum, the location of AGO1-mRNA targeting, microsomes, total polysomes, and membrane-bound polysomes, presented an increase in trimming. The importance of AGO1 membrane association for miRNA trimming and tailing was further supported by results in the mutants impaired in farnesylation transfer or in the farnesylation of J3, a protein involved in AGO1 membrane association. Altogether, these results enabled us to expose some of the complexities of miRNA turnover, with AGO1 as a central player in the pathway.

## Data Availability

All publicly available sequencing data analyzed in this study can be downloaded from public repositories using the accessions mentioned in Supplementary Table S1 and Supplementary Table S2. The data presented in this study are available as Supplementary Tables.

## Supplementary Materials

The following are available online at https://www.mdpi.com/2223-7747/10/2/267/s1, Supplementary Table S1. List of all runs analyzed with the accession of the small RNA sequencing data and additional information. Supplementary Table S2. List of all runs analyzed with the accession of the small RNA sequencing data and additional information. Supplementary Table S3. Table of miRNAs which were excluded from the analysis. Supplementary Table S4. Table of read counts for each miRNA, unmodified, trimmed, and tailed, in each sample. Supplementary Table S5. Table of miRNAs classified as guide miRNAs. Supplementary Table S6. Table of trimming and tailing indexes for each miRNA in each sample. Supplementary Table S7. Table of trimming and tailing index differences (IDs) for each miRNA in each sample. Supplementary Table S8. Table of statistical analysis.
