# Supplementary material for: Extensive Analysis of miRNA Trimming and Tailing Indicates that AGO1 Has a Complex Role in miRNA Turnover
Source: Plants (Basel). 2021 Jan 30;10(2):267. doi: 10.3390/plants10020267 (PMC7911489; doi:10.3390/plants10020267)
Supplement: Supplementary file 1 [file plants-10-00267-s001.zip › SupplementaryTableS1.docx]

| **Study** | **Ref.** | **Accession** | **Description** |
| --- | --- | --- | --- |
| Mi2008AGO1sorting | [52] | GSE10036 | Small RNA-seq from AGO1, AGO2, AGO4, AGO5 associated RNA's. WT and AGO4-Myc expressing lines in Col-0. |
| Montgomery2008AGO1 | [47] | GSE13605 | Small RNA-seq from flower tissue. WT and ago1-25 genotypes in Col-0. Soil, 16 h light+ 8 h dark. |
| Manavella2012CPL1 | [6] | ERP001622 | Small RNA-seq from 21-day-old rosette leaves. WT, cpl1-7, hyl1-2,se-3genotypes in Col-0. Soil, 16 h light+ 8 h dark. |
| Carbonell12AGO1 | [50] | GSE40259 | Small RNA-seq from AGO1-IP from flowers. wt-AGO1 (AGO1-DDH) and slicer mutant-AGO1 (AGO1-DAH) expressing plants in Col-0. Soil, 16 h light+ 8 h dark. |
| Ren2012TGH | [14] | GSE38600 | Small RNA-seq from flowers. WT and tgh-1 genotypes in Col-0. Soil,16-hour light; 8-hour dark; at 22°C. |
| Zhao12HESO1 | [26] | GSE35479 | Small RNA-seq from inflorescences. WT, heso1-1, hen1-8, heso1-1 hen1-8 genotypes in Col-0. Soil, 16 h light/ 8 h darkness at 22°C. |
| Zhai13AGO1 | [31] | GSE35562 | Small RNA-seq from flowers and seedlings. WT, hen1-1, hen1-2, ago1-11, ago1-11 hen1-2 genotypes in Ler. hen1-8 genotype in Col-0. 1/2 MS + 1% Sucrose plates under continuous light or soil in a growth chamber with 16 h of light. |
| Wang2015HENHESO1URT1 | [30] | GSE60826 | Small RNA-seq from inflorescences. WT, heso1-2, hen1-2, heso1-2 hen1-2, heso1-2 hen1-2 utr1-3 genotypes in Ler. Soil, 16 h light/ 8 h darkness at 22°C. |
| Arribas16AGO1 | [49] | PRJEB13163 | Small RNA-seq from flowes and seedlings. AGO1-IP from AGO1, AGO1E803A, AGO1H988F, AGO1D762A expressing plants in ago1-3, ago1-3 sgs3-1, ago1-3 rdr6-12. Seeds germinated in liquid  1xMurashige/Skoog medium and grown under 16h light/8h darkness regime. |
| Li2016AGO1 | [24] | GSE82041 | Small RNA-seq from 12-day seedlings (microsome fraction vs cytosol fraction). WT, ago1-27, ago1-36 genotypes in Col-0. soil, 16 h light/ 8 h darkness at 23°C. |
| Bologna2018AGO1 | [19] | GSE100395 | Small RNA-seq from AGO1-IP from inflorescences. GFP-AGO1 and GFP-AGO1ΔNES expressing plants in Col-0. Plants were grown for 2 weeks in soil in a 12-h-light/12-h-dark regimen, then moved into a 16 h/8 h chamber. |
| Derrien18AGO1 | [48] | GSE104015 | Small RNA-seq from AGO1-IP from 2-week-old seedlings. WT and ago1-57 genotypes in Col-0. 2-week-old seedlings grown on MS-agar plates. 16-h-light/8-h-dark photoperiod (22°C/20°C). |
| Sjogren18SHP40 | [51] | E-MTAB-3736 | Small RNA-seq from microsome fractionation from seedlings. WT, era1-2, J3-C417S j2-2 j3-2, wt-J3 j2-2 j3-2 genotypes in Col-0. |
| Wang2018ATRM2 | [46] | GSE107070 | Small RNA-seq from AGO1-IP from inflorescences. WT, hen1-8, hen1-8 heso1-1, hen1-8 heso1-1 atrm2-1, hen 1-8 atr2m-1, atr2m-1 genotypes in Col-0. soil, Plants were grown at 22℃ at a light intensity of approximately 120 μmol m-2 sec-1 and 16 hours light/8 hours dark photoperiod. |
| Mei2018HWS | [45] | PRJNA450254 | Small RNA-seq from inflorescences. WT, HWS OE, hws-6 genotypes in Col-0. |
| Re2019CLF | [33] | PRJEB34769 | Small RNA-seq from 16-days-old shoot apexes. WT, clf-28 genotypes in Col-0. 1/2 MS plates 16 h light + 8 h dark at 23˚C. |
| Gao2020HYL1 | [43] | PRJNA563021 | Small RNA-seq from seedlings. WT, hyl1-2 and shy43 genotypes in Col-0. Soil or 1/2 Murashige and Skoog medium at 22 °C under long-day conditions. |
| Tomassi2020CARP9 | [18] | PRJEB37499 | Small RNA-seq from 13-days-old seedlings. REPORTER LINE, hyl1-2, carp9-1 (reporter background) genotypes in Col-0. Soil, 16 h light/ 8 h darkness at 23°C. |
| Zhang2020TREX2 | [54] | GSE150473 | Small RNA-seq from inflorescences and 14-days-old seedlings. WT, thp1-5, nup1-cs, amS reporter and thp1-5 amS reporter genotypes in Col-0. Soil_16 h light+ 8 h dark at 22 ˚C or 1/2 MS with 1% Sucrose_16 h light+ 8 h dark at 22 ˚C |
